# Experiencing earthquake in the first trimester of the fetal life increases subsequent diabetes risk in the adulthood: a cross-sectional study

**DOI:** 10.1186/s12958-020-00664-2

**Published:** 2020-11-10

**Authors:** Na Li, Mei Song, Lan Wang, Xiao-chuan Zhao, Ran Wang, Yuan-yuan Gao, Lu-lu Yu, Cui-xia An, Xue-yi Wang

**Affiliations:** Department of Psychiatry, the First Hospital of Hebei Medical University, Mental Health Institute of the Hebei Medical University, No.89 Donggang Road, Yuhua District, Shijiazhuang, 050031 Hebei China

**Keywords:** Prenatal earthquake stress, Middle age, Diabetes mellitus, Risk factors

## Abstract

**Objective:**

To investigate the long-term effect of prenatal exposure to earthquake stress on diabetes risk in the adulthood.

**Methods:**

This study included employees of Tangshan Kailuan Mining Group between July 29, 1976 and April 28, 1977. The exposure group included subjects who experienced the Tangshan Earthquake during their prenatal period and who had lived in Tangshan since birth. The non-exposure group included subjects who were born 1–1.9 years after the earthquake and who had lived in Tangshan since birth. A questionnaire was designed that included sociodemographic information, conditions during pregnancy, and earthquake experience. Anthropometric measurements including height and weight, body mass index (BMI), waist circumference were made. Fasting plasma glucose (FPG) and lipid profiles were also determined.

**Results:**

Totally 947 subjects were included with 397 subjects in the exposed group and 550 subjects in the non-exposed group. The diabetes rate is significant different in these four groups(*χ*^2^ =8.045, *P* = 0.045). Moreover, 11.8, 7.5 and 8.0% of the subjects who were exposed to earthquake in the 1st, 2nd, and 3rd trimester of pregnancy had diabetes. 5.1% of the subjects had diabetes in non-exposure group. Our multivariate analysis showed that 1st trimester (OR 2.481, 95%CI 1.02, 6.034; *P* = 0.045) and loss of family members during earthquake (OR 2.452, 95%CI 1.293, 4.653; *P* = 0.006) were associated with significantly increased risk of diabetes.

**Conclusions:**

Exposure to earthquake during the first trimester of pregnancy and experience of family member loss in the earthquake significantly increased the subsequent risk of diabetes in the middle age (36–39 years of age). Our data suggest that earthquake experience in the early pregnancy has a longer-term effect on diabetes risk during adulthood.

## Introduction

The maternal pregnancy (prenatal stage) is an important period for the healthy development of the fetus. However, stressful life events during pregnancy not only impact on the mother and fetal development in utero but also cause long-term adverse outcomes in the offspring born from stress-burdened mothers [1, 2]. Increasing evidence has indicated that the origins of certain age-related diseases such as diabetes could be traced back to stressful experiences during the fetal period in utero [3]*.* Stress can change blood flow to the uterus and hence to the fetus, and may cause structural and/or functional alterations in the cell, tissue, and organ system of the fetus. The incidence of diabetes mellitus was reportedly increased by 22% in children exposed to hurricane during their infancy or prenatal life [4].

During the fetal period, the brain regions that are involved in modulating the hypothalamus-pituitary-adrenal (HPA) axis, such as the hippocampus, amygdala, frontal cortex, have not been completely developed and are susceptible to stressful stimuli. Studies on earthquake experience confirmed that earthquake trauma could increase the risk of diabetes mellitus. Ciocca et al. found that hyperglycemia was more common in patients who developed post-traumatic stress disorder (PTSD) after 2009 L’Aquila in Italy [5]. After the Eastern Japan earthquake on 11th March 2011, a study of 497 diabetic patients showed that blood glucose control was poor in 3 months after the earthquake, which was related to endogenous insulin secretion caused by hyperactivity of sympathetic nerve caused by stress [6]. Our previous study found that the resting heart rate and fasting blood glucose level increased significantly in adults who experienced earthquake stress during their adolescence and childhood [7, 8]. However, there has been no study on the subsequent risk of diabetes in adults who have experienced earthquake in their prenatal life.

On the early morning of July 28, 1976, an earthquake with a magnitude of 7.8 hit Tangshan, Hebei province, China and lasted to 14–16 s and followed by a major 7.1 magnitude aftershock. Approximately a quarter million people succumbed and 160.000 people suffered from severe injuries. Such catastrophic earthquake could cause extreme stress on pregnant mother and their developing fetuses. In the current cross-sectional study, we sought to determine the prevalence of diabetes mellitus among adults who had experienced the 1976 Tangshan Earthquake in their fetal life.

## Subjects and methods

### The study population

This study included employees of Tangshan Kailuan Mining Group between July 29, 1976 and April 28, 1977. The exposure group included subjects who experienced the Tangshan Earthquake during their prenatal period and who had lived in Tangshan since birth. The non-exposure group included subjects who did not experience the Tangshan Earthquake during their fetal period and were born 1–1.9 years after the earthquake and who had lived in Tangshan since birth. Exclusion criteria were as follows: 1) acute disease or traumatic surgery; 2) severe liver disease; 3) secondary diabetes mellitus (such as pancreatic disease or pancreatectomy, Cushing’s syndrome, and thyroid disease, etc.); 4) psychiatric diseases; 5) pregnant women; 6) history of severe stressful life events.

The study protocol was approved by the Ethics Committee of the First Hospital of Hebei Medical University (No 2014005). Informed consents were obtained from all study subjects before enrollment.

### Questionnaire

A questionnaire was designed that included sociodemographic information, conditions during pregnancy, and earthquake experience. Information regarding the earthquake and pregnancy was provided by parents of the study subjects. Demographic information included age, gender, education, marriage, and monthly income. Earthquake experience included mother’s condition, infant’s birth weight and loss of any family members.

Past history included past physical and mental illness, smoking and drinking. Smoking was defined as a continuous or cumulative smoking for more than 6 months in a lifetime, with at least 1 cigarette per day. Smoking cessation or abstinence: those who met smoking or drinking conditions had been at least 6 months without smoking or drinking at the time of investigation. Alcohol consumption was defined as an average daily alcohol content of 100 mL (alcohol content was more than 50%), lasting more than 6 months. Light alcohol consumption was defined as an average monthly alcohol consumption less than 120 standard cups [9]. Heavy alcohol consumption was defined as an average daily alcohol consumption above 50 mL (about 4 standard cups) or an average monthly alcohol consumption of more than 120 standard cups.

### Physical examination

Height and weight were measured by the corrected RGZ-120 body weight scale. Body weight was measured with light clothes on and shoes and hats taken off. Body mass index (BMI) was calculated as weight divided by height squared (Kg/m^2^). Waistline measurement was the circumference of the finest position of the waist, with the value was accurate to 0.1 cm. Blood pressure was measured 30 min after the subject had stopped smoking, drinking tea or coffee. The right arm brachial artery blood pressure was measured by the corrected mercury sphygmomanometer. Three measurements were made at an interval of 1–2 min and the average value was adopted. Hypertension was diagnosed by systolic blood pressure (SBP) ≥ 140 mmHg and/or diastolic blood pressure (DBP) ≥ 90 mmHg, or subjects were taking the antihypertensive medications at the time of the study. Diabetes mellitus was diagnosed if FPG was > 126 mg/dL (7.0 mmol/L), or subjects who were taking hypoglycemic drugs or using insulin at the time of the study.

### Laboratory study

Blood samples were collected in 5 mL EDTA vacuum collection tubes (Inspeck, ST750EK, Sekisui, Osaka, Japan) between 7:00 a.m. and 9:00 a.m.. Blood samples were centrifuged at 3000×g for 10 min at room temperature, and the supernatant was collected for assaying fasting plasma glucose (FPG), triglycerides (TG), low-density lipoprotein cholesterol (LDL-C), total cholesterol (TC), and high-density lipoprotein cholesterol (HDL-C). FPG levels were tested by the hexokinase method (BioSino Bio-Technology & Science Inc.) TG, LDL-C, HDL-C and TC were measured by the oxidase method (Shanghai Mind Bioengineering Co, Ltd., Shanghai, China). Measurements were performed using a Hitachi 7600 Automatic Biochemical Analyzer.

### Statistical analysis

All data were analyzed using SPSS21.0 (SPSS Inc., Chicago, IL, USA). The exposure group was further divided into the first trimester group for subjects who were (born between January 29, 1977 and April 28, 1977), the second trimester group for subjects who were born between October 29, 1976 and January 28, 1977) and the third trimester group who were born between July 29, 1976 and October 1976. One-way ANOVA was used to compare variables among groups. Kruskal-Wallis test and Student’s *t* test were used for comparison of continuous variables between subjects in the exposed and non-exposed group where appropriate. The comparison of the prevalence of hypertension and diabetes mellitus among groups was performed using the chi-square test. We used multivariate logistic regression analysis in which the incidence of diabetes was used as a dependent variable while hypertension, gender, age, earthquake stress during different gestational periods, BMI, TC, TG, HDL, LDL, family history of diabetes, family loss, smoking and drinking status were used as independent variables. *P* < 0.05 was considered statistically significantly different.

## Results

### Demographic and baseline characteristic of the study population

Totally 1081 subjects met the inclusion criteria and returned the questionnaires. Nine hundred forty-seven (87.6%) of the questionnaires were effective. They included 397 subjects in the exposed group and 550 subjects in the non-exposed group. The demographic and baseline characteristic of the study population are shown in Table 1. The two groups were comparable in demographic and baseline variables (*P* > 0.05) except age (*χ*^2^ =411.82, *P* < 0.01).

**Table 1:** Sociodemographic characteristics of the study population

	Exposure to earthquake			Non-exposure to earthquake	*χ*^2^	***P***
	3rd trimester	2nd trimester	1st trimester			
**N**	137	133	127	550		
**Male gender, n(%)**	125 (91.2)	116 (87.2)	107 (84.3)	476 (86.5)	3.135	0.375
**Ethnicity, n(%)**					1.208	0.751
Han	136 (99.3)	130 (97.7)	125 (98.4)	539 (98.0)		
Minorities	1 (0.7)	3 (2.3)	2 (1.6)	11 (2.0)		
**Educational level, n(%)**					8.628	0.196
< 6 years	3 (2.2)	3 (2.3)	2 (1.6)	6 (1.1)		
6–12 years	103 (75.7)	94 (70.7)	87 (69.6)	361 (65.6)		
> 12 years	30 (22.1)	36 (27.1)	36 (28.8)	183 (33.3)		
**Mean age, years**	39.0 ± 0.2	38.7 ± 0.5	38 ± 0.3	37.5 ± 0.6	411.82	< 0.0001
**Marital status**					4.147	0.657
Not married	3 (2.2)	2 (1.5)	2 (1.6)	3 (0.5)		
Married	126 (93.6)	125 (94)	119 (93.7)	515 (93.6)		
Divorce, remarriage and widowhood	8 (5.8)	6 (4.5)	6 (4.7)	32 (5.8)		
**Occupation, n(%)**					1.810	0.575
Manual labor	111 (84.7)	112 (87.5)	101 (82.1)	438 (82.2)		
Others	26 (15.3)	21 (12.5)	25 (17.9)	115 (17.8)		
**Smoking history, n(%)**					3.413	0.756
Smoker	68 (49.6)	65 (49.2)	61 (48.8)	277 (50.9)		
Former smoker	15 (10.9)	10 (7.6)	8 (6.4)	54 (9.9)		
Non-smoker	54 (39.4)	57 (43.2)	56 (44.8)	213 (39.2)		
**Drinking, n(%)**					4.008	0.261
No drinking	36 (26.3)	45 (33.8)	40 (31.5)	150 (27.3)		
Light drinking	84 (61.3)	77 (57.9)	81 (63.8)	369 (67.1)		
Heavy drinking	17 (12.4)	11 (8.3)	6 (4.7)	31 (5.6)		
**Family loss in the earthquake, n(%)**	65 (47.4)	62 (46.6)	247 (44.9)	55 (43.3)	0.585	0.900
**Diabetes mellitus, n(%)**	11 (8.0)	10 (7.5)	15 (11.8)	28 (5.1)	8.045	0.045

### Anthropometric and metabolic characteristics of the study population

The anthropometric and metabolic characteristics of the study population are shown in Table 2. The average of SBP in the third trimester group (123.6 ± 9.7 mmHg) and the second trimester group (123.5 ± 11.0 mmHg) were higher than that of the non-exposure group (121.3 ± 12.4 mmHg). The average of FBG in the third trimester group (5.12 ± 2.63 mmHg), the second trimester group (5.10 ± 1.22 mmHg) and the first trimester group (5.20 ± 1.33 mmHg) were higher than that of the non-exposure group (4.96 ± 1.35 mmHg).

**Table 2:** Anthropometric and metabolic characteristics of the study population (mean ± SD) or median(P25, P75)

	Exposure to earthquake		No exposure	
	3rd trimester	2nd trimester	1st trimester	
N	137	133	127	
BMI (kg/m^2^)	25.55 ± 3.14	24.82 ± 2.84	24.88 ± 3.10	25.11 ± 3.16
SBP (mmHg)	123.6 ± 9.7	123.5 ± 11.0	121.9 ± 10.6	121.3 ± 12.4
DBP (mmHg)	81.7 ± 8.4	81.1 ± 8.9	81.1 ± 7.3	83.2 ± 9.4
FPG (mmol/L)	5.12 ± 2.63	5.10 ± 1.22	5.20 ± 1.33	4.96 ± 1.35
TC (mmol/L)	5.26 ± 1.15	5.20 ± 0.98	5.06 ± 0.96	5.09 ± 1.09
TG (mmol/L)	3.0 (1.5,5.6)	2.1 (1.1,2.8)	1.5 (0.8,2.9)	2.2 (1.2,3.3)
HDL (mmol/L)	1.62 ± 0.31	1.67 ± 0.35	1.63 ± 0.36	1.64 ± 0.34
LDL (mmol/L)	2.80 ± 0.74	2.87 ± 0.72	2.83 ± 0.67	2.84 ± 0.76

Note. *BMI* body mass index, *SBP* systolic blood pressure, *DBP* diastolic blood pressure, *FPG* fasting plasma glucose, *TC* total cholesterol, *TG* triglyceride, *HDL* high density lipoprotein, *LDL* low density lipoprotein

### Incidence of diabetes mellitus

The diabetes rate is significant different in these four groups(*χ*^2^ =8.045, *P* = 0.045). Moreover, 11.8, 7.5 and 8.0% of the subjects who were exposed to earthquake in the 1st, 2nd, and 3rd trimester of pregnancy had diabetes. 5.1% of the subjects had diabetes in non-exposure group.

### Multivariate analysis of risk factors for diabetes

Our multivariate analysis showed that 1st trimester (OR 2.481, 95%CI 1.02,6.034; *P* = 0.045) and loss of family members during earthquake (OR 2.452, 95%CI 1.293,4.653; *P* = 0.006) were associated with significantly increased risk of diabetes. In addition, higher BMI (OR 1.113, 95%CI 1.004,1.233; *P* = 0.042), higher TG levels (OR 1.194, 95%CI 1.045,1.364; *P* = 0.009) were independent risk factors for diabetes. Meanwhile, light alcohol consumption (OR 0.181, 95%CI 0.055,0.597; *P* = 0.005) was associated with reduced risk of diabetes (Table 3).

**Table 3:** Multivariate logistic regression analysis of risk factors of diabetes mellitus

	B	S.E	Walds	***P***	***OR***	***95%CI***
Hypertension	−0.132	0.383	0.118	0.731	0.877	(0.414,1.858)
Gender	−1.509	0.792	3.634	0.057	0.221	(0.047,1.043)
Age	0.149	0.348	0.184	0.668	1.161	(0.587,2.296)
BMI	0.107	0.053	4.122	0.042	1.113	(1.004,1.233)
TC	−0.011	0.201	0.003	0.958	0.99	(0.667,1.467)
TG	0.177	0.068	6.817	0.009	1.194	(1.045,1.364)
HDL	0.375	0.478	0.615	0.433	1.455	(0.57,3.716)
LDL	0.391	0.224	3.062	0.08	1.479	(0.954,2.293)
Sub-groups			4.974	0.174		
3rd trimester	0.893	0.623	2.056	0.152	2.443	(0.72,8.281)
2nd trimester	1.03	0.574	3.215	0.073	2.8	(0.909,8.63)
1st trimester	0.909	0.453	4.014	0.045	2.481	(1.02,6.034)
Family history of diabetes	0.626	0.458	1.866	0.172	1.869	(0.762,4.587)
Family loss	0.897	0.327	7.537	0.006	2.452	(1.293,4.653)
Smoking status						
Non- smoker			3.165	0.205		
Light smoker	0.499	0.363	1.892	0.169	1.647	(0.809,3.352)
Heavy smoker	0.886	0.538	2.714	0.1	2.425	(0.845,6.959)
Drinking status						
Non-drinker			13.455	0.004		
Light drinker	−1.715	0.54	10.097	0.005	0.181	(0.055,0.597)
Heavy drinker	1.101	0.583	3.568	0.059	3.007	(0.959,9.426)
Constant	−12.379	13.088	0.895	0.344	0	

Note. *BMI* body mass index, *TC* total cholesterol, *TG* triglyceride, *HDL* high density lipoprotein, *LDL* low density lipoprotein

## Discussion

Our study found that exposure to earthquake during the first trimester of pregnancy and experience of family member loss in the earthquake significantly increased the subsequent risk of diabetes in the middle age (36–39 years of age). Our data suggest that earthquake experience in the early pregnancy has a longer-term effect on diabetes risk during adulthood.

Previous studies have shown that maternal mental distress during the critical period of fetal development could impact on the neuroendocrine function of offspring [4, 10]. Animal experiments also confirmed that maternal stressful events during pregnancy had significant effects on behavioral and metabolic phenotypes in the adult offspring of rats [11, 12]. Mental trauma could lead to increased blood glucose, which is mainly due to the maladaptive pattern of stress-related hormones. The HPA axis would become overactive and release excessive amounts of cortisol and other hormones when an individual is exposed to traumatic events [13, 14], which could lead to obesity, massive visceral fat accumulation, dyslipidemia and increased insulin resistance [15]. On the other hand, increased glucocorticoid during pregnancy might change the responsiveness toward stress and reduces the expression of 11β-hydroxysteroid dehydrogenase, and finally increase anxiety like behaviors and decrease neurodevelopment of offspring. This could increase vulnerability to hypertension, dyslipidemia, hyperglycemia and metabolic-related diseases of middle-aged persons [16]. In the present study, women suffered from severe earthquake trauma during pregnancy, suggesting that dramatic changes in the internal environment could give rise to sympathetic hyperfunction, which plays an important role in intrauterine vasoconstriction and fetal hypoxia. Meanwhile, the excessive release of stress-related hormones from the maternal body to the fetus via the placenta could lead to an increased risk of compromised fetal development and metabolic abnormalities.

A large epidemiological survey of 408,015 Dutch men born in 1944–1947 confirmed that there was a significant correlation between famine exposure in the first trimester of pregnancy and the long-term mortality of offspring, suggesting that early-life stressful events influenced the outcome of chronic diseases [17]. This is in line with the results of our research. The first trimester of pregnancy is a critical period for the formation of the fetal organs, which is very sensitive to the stimulation of the internal and external environment during this period. At this time, stress could substantially increase the subsequent risk of somatic disease.

A study of maternal exposure to the famine in Holland found that with an increase of blood glucose levels by 0.3 mmol/L, the mean waist circumference increased by 3 cm, and the risk of obesity (BMI > 25 kg/m^2^) increased significantly. In our study, the level of FPG increased by 0.3 mmol/L in the early stage of pregnancy than in the non-exposure group [16]. Our study did not find a significant correlation on mid-age BMI and maternal exposure to earthquake, which might be due to different stressors, and our subjects were all coal miners who mostly engaged in heavy physical labor.

Our present study found that the loss of relatives increased the risk of long-term diabetes in the first trimester. Previous studies found that after experiencing major life events such as death of the eldest son or a loved one, the subsequent risk of type 2 diabetes increased significantly [18]. Other surveys found that the prevalence of diabetes increased significantly in the offspring of pregnant women when their mothers passed away from their loved ones or family members. The sudden loss of kinship caused by the earthquake, especially the loss of first-degree relatives, could significantly increase the stress caused by the earthquake itself, increase the intensity and duration of stress of pregnant women, thereby directly and indirectly affecting the development of the fetus and increasing the risk of metabolic diseases of the offspring.

Our study didn’t find a correlation between smoking and heavy drinking and the development of diabetes, while light alcohol consumption was a protective factor for diabetes. Wakabayashi et al. investigated 12,627 middle-aged Japanese men and explored the risk of alcohol consumption on diabetes. Compared with non-drinkers, light-to-moderate alcohol consumption (< 22 g/day) could significantly reduce the risk of diabetes, while extremely heavy drinking (> 44 g/day) had no significant impact on diabetes [19]. A meta-analysis, which included 13 prospective studies, showed that alcohol consumption (g/day) was associated with the prevalence of diabetes into the U-curve, and that drinking alcohol or beer per day for 20–30 g/days was the lowest in diabetes. This is basically the same as our research results [14].

There were some limitations in our present study. Given the cross-sectional design and relatively small female sample cohort of the study, it was relatively less representative. A further follow-up was required to clarify the influence of earthquake stress on psychosomatic diseases. In addition, there were many confounding factors in psychosomatic disease, such as diet habits, lifestyle and family status (e.g. single-parent family, economic status, and parenting style, etc.); the above factors, however, are difficult to quantify at present. Moreover, our study will further explore the mechanism of prenatal stress and psychosomatic diseases through gene polymorphism, DNA methylation and multimodal fMRI.

In conclusion, experience of earthquake during the first trimester of the fetal life and loss of family member loss in the earthquake significantly increased the subsequent risk of diabetes in the adulthood, suggesting that earthquake experience in the early pregnancy has a longer-term effect on diabetes risk.

## Data Availability

The datasets generated and analyzed during the current study are available from the corresponding author on reasonable request.

## Abbreviations

BMI: Body mass index
FPG: Fasting plasma glucose
HPA: Hypothalamus-pituitary-adrenal
PTSD: Post-traumatic stress disorder
SBP: Systolic blood pressure
DBP: Diastolic blood pressure
TG: Triglycerides
LDL-C: Low-density lipoprotein cholesterol
TC: Total cholesterol
HDL-C: High-density lipoprotein cholesterol
